# Women’s perception of quality of maternity services: a longitudinal survey in Nepal

**DOI:** 10.1186/1471-2393-14-45

**Published:** 2014-01-24

**Authors:** Rajendra Karkee, Andy H Lee, Paras K Pokharel

**Affiliations:** 1 School of Public Health and Community Medicine, BP Koirala Institute of Health Sciences, Dharan, Nepal; 2 School of Public Health, Curtin University, Perth, WA, Australia

**Keywords:** Quality of care, Maternity services, Perceptions, Nepal

## Abstract

**Background:**

In the context of maternity service, the mother’s assessment of quality is central because emotional, cultural and respectful supports are vital during labour and the delivery process. This study compared client-perceived quality of maternity services between birth centres, public and private hospitals in a central hills district of Nepal.

**Methods:**

A cohort of 701 pregnant women of 5 months or more gestational age were recruited and interviewed, followed by another interview within 45 days of delivery. Perception of quality was measured by a 20-item scale with three sub-scales: health facility, health care delivery, and interpersonal aspects. Perceived quality scores were analysed by ANOVA with post-hoc comparisons and multiple linear regression.

**Results:**

Within the health facility sub-scale, birth centre was rated lowest on items ‘adequacy of medical equipment’, ‘health staff suited to women’s health’ and ‘adequacy of health staff’, whereas public hospital was rated the lowest with respect to ‘adequacy of room’, ‘adequacy of water’, ‘environment clean’, ‘privacy’ and ‘adequacy of information’. Mean scores of total quality and sub-scales health facility and health care delivery for women attending private hospital were higher (p < 0.001) than those using birth centre or public hospital. Mean score of the sub-scale interpersonal aspects for public hospital users was lower (p < 0.001) than those delivered at private hospital and birth centre. However, perception on interpersonal aspects by women using public hospital improved significantly after delivery (p < 0.001).

**Conclusions:**

Overall, perception of quality differed significantly by types of health facility used for delivery. They rated lowest the supplies and equipment in birth centres and the amenities and interpersonal aspects in the public hospital. Accordingly, attention to these aspects is needed to improve the quality.

## Background

Quality of care is an important but often neglected issue in safe motherhood programmes [1]. Quality of care can be considered from the provider or user’s perspective, and is differentiated into observed and perceived quality [2,3]. Users, in reality, play a central role in defining and assessing quality of care because they choose whether or where to go for care based on their opinions, previous experiences with the health system and those of people they know [4,5]. Perception of low quality has been reported as a major factor in non-utilisation or bypassing of health services [6-11].

In accessing obstetric care, women can be influenced by health system factors, such as a respectful provider attitude, competency, and availability of drugs and medical equipment [12]. Cultural inappropriateness of care, disrespectful and inhumane services, and lack of emotional support, can deter them from accessing obstetric care [13-16]. On the other hand, positive client perception of doctor and nurse skills can increase utilisation of delivery services [17,18]. Support in the form of comfort, reassurance and praise during childbirth is particularly beneficial [19].

Indeed, the mother’s assessment of quality is central because emotional, cultural and respectful supports are needed during labour and the delivery process [3,20]. Women and their family often decide the location for childbirth based on their opinions, evaluations and experience with the maternity services [21]. Perceptions of higher technical quality attract women to deliver at hospitals, unlike health centres which typically cannot provide emergency operation and lack competent midwives and doctors [22-25]. Therefore, client-perceived quality is an important issue in delivery service utilisation [26-28].

Perceived quality of care can be assessed subjectively using a qualitative approach, such as suggestion boxes to obtain feedback, participant or non-participant observation, analysis of formal complaints or through satisfaction surveys [29,30]. Such methods have been criticised due to reporting biases, inability to trap consumers’ relative preferences for different attributes of quality, and they tend to be difficult to interpret, with little evidence of reliability or validity [31]. In recent years, perceived quality is quantified objectively using standardised and validated instruments that collect information from the users, who rate the quality of service structure, the actual healthcare activities and outcomes [2,31-33]. The information so obtained is directly interpretable and actionable to improve the quality.

Few studies of user perspectives of healthcare services have been undertaken in Nepal. The quality factors that are pertinent to public hospital clients include attitude, interpersonal and technical skills of service personnel [34]. Health facilities in Nepal are often understaffed with poor infrastructure, equipment and management [35,36]. An assessment of quality of care in midwifery and emergency obstetric care services in Nepal found major shortfalls [37]. However, client-perceived quality of maternity services has never been systematically assessed in Nepal. The aim of the present study was to compare the perceived quality of maternity services between birth centres, public and private hospitals in Nepal. Such assessment and comparison of perceived quality will identify strength and weakness of the maternity services, and can assist in quality improvement by making services more responsive and client-oriented.

## Methods

### Study location and population

Kaski is a central hill district of Nepal with 82% literacy and 13800 expected annual pregnancies [38]. The district has a central valley, with Pokhara being the second largest city in Nepal, and rest of the land spreads out into hilly terraces. The Pokhara city is the location of the Western Regional Government Hospital (hereafter referred as the ‘public hospital’) and two teaching hospitals of private medical colleges (hereafter referred as the ‘private hospitals’). The three hospitals provide free maternity services and serve as referral centres for emergency obstetric care.

The rural part of the district is divided into 13 *illakas*. In each rural *illaka*, at least one primary health care centre or health post has been upgraded to a ‘birth centre’, which has skilled birth attendants (midwives or trained auxiliary nurses) to provide free delivery and basic emergency obstetric services. The rural areas are connected with the central valley via non-gravelled roads.

A large community-based prospective cohort study of maternity service utilisation was recently conducted between 1 December 2011 and 30 October 2012. A total of 701 pregnant women with 5 months or more gestation were recruited from the urban and rural areas of the Kaski district. Details of the sampling procedure and delivery location have been described elsewhere [39,40]. The present study focused on 547 postpartum women from the initial cohort who delivered in a healthcare facility. Participants excluded were those delivered at home (n = 92), delivered on the way to a facility (n = 5), lost to follow up (n = 43), had antepartum stillbirths (n = 9), had intrapartum stillbirths (n = 3) or early neonatal deaths after home delivery (n = 2). Women who experienced stillbirths or had early neonatal deaths were excluded due to ethical reasons as it would be inappropriate to interview them after their tragic loss.

### Data collection

The instrument for assessing perceived quality of maternity services was based on a validated 20-item scale developed for Vietnam [33]. It was tested on 25 postpartum women for suitability in the study area. The scale was subsequently adjusted to match local understanding, maternity service provision and special aspects of maternity care in the Nepalese context [20,41]. Items related to economic accessibility were removed because all maternity services in the Kaski district were free under ‘safer mother programme’ of the Nepalese government [40].

The final scale consisted of 20 items in three dimensions: 7 items on health facility, 8 items on health care delivery, and 5 items on interpersonal aspects of health care; see Additional file 1: Table S1. Cronbach’s alpha confirmed internal consistency of the scale, which was 0.85 for the subscale health facility, 0.65 for the subscale health care delivery, 0.62 for the subscale interpersonal aspect, and 0.85 for the whole scale. Concurrent validity was evident with correlation of 0.78 between the total perceived quality score and overall patient satisfaction score (as measured by a four-point Likert scale).

Baseline information on socio-demographic characteristics and perceived quality of nearest government health facility (birth centre for rural women and public hospital for urban women) was collected at pregnancy stage by 15 local female data collectors between 1 December 2011 and 31 January 2012. The same data collectors then followed up and visited the house of participants within 45 days postpartum. These interviewers assessed the perceived quality of facility where the women actually delivered, by reading out statements related to each item of the perceived quality scale in the national ‘Nepali’ language. Respondents expressed their opinion or experience on a four-point Likert scale: completely disagree (1), disagree (2), agree (3), and completely agree (4). A pictorial diagram of four emotional (happy and weeping) faces was shown to facilitate rating on the level of agreement.

### Statistical analysis

Table 1 lists the client-related variables. An asset score for socioeconomic status was generated from the first component of a principal components analysis [42], utilising survey questions on household assets. This asset score was then used to develop wealth quintiles. Distance to the health facility where women delivered was estimated by the time took on foot or by vehicle to reach the facility (≤ 30 min; > 30 min). Education level was recorded as none or primary, and secondary or above. Caste was classified according to the government’s health system: upper caste, lower caste, janajati and religious minorities. ‘Upper caste’ and ‘lower caste’ refer to Indo-Aryan people, whereas ‘janajati’ refers to Tibeto-Burman people. The term ‘religious minorities’ denotes mainly Muslims and Christians. Only three respondents belonged to religious minorities and were merged with the janajati group.

**Table 1:** Characteristics of participants by type of health facility (n = 547), Kaski district, Nepal, 2012

**Characteristic**	**Birth centre**	**Public hospital**	**Private hospital**	**Total**	P*
	**n (%)**	**n (%)**	**n (%)**	**n (%)**	
n	77	419	51	547	
Age (years)					0.23
15-19	14 (18.1)	55 (13.1)	7 (13.7)	76 (13.9)	
20-24	41 (53.2)	217 (51.8)	21 (41.2)	279 (51.0)	
25-40	22 (28.6)	147 (35.1)	23 (45.0)	192 (35.1)	
Parity					0.43
Primipara	37 (48.0)	230 (54.9)	24 (47.0)	292 (53.4)	
Multipara	40 (52.0)	189 (45.1)	27 (53.0)	255 (46.6)	
Wealth					<0.001
Lowest quintile	46 (60.0)	46 (11.0)	6 (11.8)	99 (18.2)	
Other quintiles	31 (40.0)	371 (89.0)	45 (88.2)	446 (81.8)	
Caste					0.02
Upper caste	36 (46.7)	235 (56.3)	30 (58.8)	301 (55.2)	
Janajati	13 (16.8)	100 (24.0)	12 (23.5)	125 (22.9)	
Lower caste	28 (36.4)	82 (19.7)	9 (17.7)	119 (21.8)	
Education level					0.16
None to primary	25 (32.5)	102 (24.3)	11 (21.5)	138 (25.2)	
Secondary and above	52 (67.5)	317 (75.7)	40 (78.4 )	409 (74.8)	
Distance to reach facility					<0.001
≤ 30 min	22 (29.5)	254 (60.6)	31 (60.7)	307 (56.1)	
> 30 min	55 (71.4)	165 (39.4)	20 (39.2)	240 (43.9)	

*Chi-square test of association with facility type.

The outcome variable was client-perceived quality. Perceived quality scores were compared by ANOVA with Tukey’s post-hoc comparisons between women who delivered at birth centre, public hospital and private hospital. Paired t-tests were used to ascertain changes in perceived quality scores within the subgroup of women who did not change the type of facility from antenatal visit to actual delivery. Further, multiple linear regressions were conducted to assess differences on perceived quality while accounting for the effects of client characteristics. All statistical analyses were performed using SPSS version 18 [43].

The project protocol was approved by the Human Research Ethics Committee of Curtin University (approval number HR 130/2011), Ethical Review Board of Nepal Health Research Council (approval number 88/2011) and the District Public Health Office of Kaski. An information sheet was provided and read to each participant before obtaining her signed or thumb-print informed consent.

## Results

### Respondent characteristics

Of the 547 women in the cohort, 77 delivered at birth centres, 419 at public hospital and 51 at private hospitals. The number of deliveries at birth centres was low because many rural women bypassed their local birth centres to deliver at urban hospitals [44]. As shown in Table 1, more than half the respondents were primipara (53.4%), belonged to upper caste (55.2%), received secondary or above education (74.8%), and lived within half an hour from the health facility (56.1%). Women who delivered at birth centres were poorer, belonged to low caste and lived far away from facility when compared to their counterparts who delivered at hospitals.

### Perceived quality of the health facilities

Table 2 compares perceived quality between the three facilities. Within the health facility sub-scale, birth centres were rated lowest on items ‘adequacy of medical equipment’, ‘health staff suited to women’s health’ and ‘adequacy of health staff’, whereas public hospital was rated the lowest with respect to ‘adequacy of rooms’, ‘adequacy of water’, ‘environment clean’, ‘privacy’ and ‘adequacy of information’.

**Table 2:** Perceived quality item scores by type of health facility, Kaski district, Nepal, 2012

**Item**	**Birth centre (n = 77)**	**Public hospital (n = 419)**	**Private hospital (n = 51)**	P*
	**Mean (SD)**	**Mean (SD)**	**Mean (SD)**	
**Health facility**	19.37 (3.50)	19.35 (3.20)	23.14 (3.43)	<0.001
(min 7, max 28)				
Adequacy of health staff	2.75 (0.64)	3.14 (0.61)	3.38 (0.53)	
Health staff suited to women’s health	2.69 (0.68)	3.10 (0.60)	3.40 (0.49)	
Adequacy of room	3.01 (0.49)	2.54 (0.70)	3.32 (0.55)	
Adequacy of water	2.76 (0.78)	2.44 (0.71)	3.30 (0.58)	
Environment clean	2.84 (0.62)	2.41 (0.70)	3.36 (0.56)	
Adequacy of medical equipment	2.53 (0.63)	2.95 (0.58)	3.38 (0.56)	
Distance	2.78 (0.61)	2.75 (0.59)	3.00 (0.69)	
**Health care delivery**	23.01 (2.03)	23.42 (3.22)	25.88 (3.61)	<0.001
(min 8, max 32)				
Examine well	2.88 (0.35)	3.02 (0.62)	3.34 (0.59)	
Staff capable	2.83 (0.37)	3.00 (0.56)	3.36 (0.52)	
Prescription of drugs	2.96 (0.29)	2.97 (0.54)	3.30 (0.54)	
Quality of drugs	2.84 (0.48)	3.00 (0.52)	3.26 (0.52)	
Availability of drugs	2.77 (0.52)	2.87 (0.58)	3.12 (0.65)	
Privacy	2.76 (0.53)	2.68 (0.66)	3.16 (0.61)	
Unnecessary act	3.07 (0.35)	3.02 (0.30)	3.06 (0.23)	
Adequacy of information	2.85 (0.52)	2.82 (0.61)	3.28 (0.53)	
**Interpersonal aspects**	14.55 (1.78)	13.27 (2.60)	15.36 (2.38)	<0.001
(min 5, max 20)				
Openness to patients	2.87 (0.49)	2.64 (0.65)	3.08 (0.60)	
Compassion for patients	2.91 (0.46)	2.60 (0.64)	3.00 (0.57)	
Respect for patients	2.96 (0.33)	2.56 (0.61)	2.98 (0.62)	
Time devoted to patients	2.94 (0.31)	2.69 (0.56)	3.22 (0.54)	
Honesty	2.85 (0.50)	2.77 (0.53)	3.08 (0.52)	
**Total score**	56.93 (6.73)	56.04 (7.86)	64.38 (8.55)	<0.001
(min 20, max 80)				

*ANOVA test of differences in mean scores between health facilities.

The total perceived score and the three sub-scale scores differed significantly between types of health facility used for delivery based on Tukey’s post hoc tests; details are presented in Additional file 2: Table S2. Mean scores of total quality and sub-scales health facility and health care delivery for women attending private hospital were higher (p < 0.001) than those using birth centre or public hospital. Mean score of the sub-scale interpersonal aspects for public hospital users was lower (p < 0.001) than those delivered at private hospital and birth centre. No significant difference was found in health facility and health care delivery subscale mean scores between the birth centre and the public hospital groups. Similarly, there was no significant difference in mean score of interpersonal aspect between birth centre and private hospital groups. The observed differences on perceived quality between facilities remained significant after adjusting for the effects of socio-demographic and client-related variables by multiple regression analysis; results of which are provided in Additional file 3: Table S3.

### Perceived quality before and after delivery

Table 3 compares perceived quality before and after delivery for the subgroup of women who did not change the type of facility from antenatal visit to actual delivery. While perception of quality did not change pre- and post- delivery for the birth centre group, improvement in perceived quality of the public hospital was observed after delivery, especially on interpersonal aspects (p < 0.001).

**Table 3:** Comparison of perceived quality before and after delivery at the same facility

**Perceived quality of government health facility**	**Before delivery mean (SD)**	**After delivery mean (SD)**	P*
**Birth centre (n = 77)**			
Health facility	19.72 (2.08)	19.36 (3.52)	0.40
Health care delivery	23.35 (2.08)	23.05 (2.01)	0.27
Interpersonal aspects	14 (2.05)	14.54 (1.79)	0.07
Total score	57.09 (4.97)	56.96 (6.77)	0.87
**Public hospital (n = 275)**			
Health facility	19.57 (3.36)	19.93 (3.31)	0.12
Health care delivery	23.40 (3.71)	23.79 (3.56)	0.10
Interpersonal aspects	12.19 (2.65)	13.32 (2.75)	<0.001
Total score	55.17 (8.21)	57.04 (8.44)	<0.001

*Paired *t*-test of differences in mean scores before and after delivery.

## Discussion

This large community-based longitudinal study found significant differences in perceived quality of maternity services by type of health care facilities used for delivery, even when client-related characteristics were taken into account. Our finding was consistent with a previous study in Burkina Faso [45]. In particular, private hospitals were perceived of higher quality for maternity services. The literature has also shown that the private sector outperforms the public sector in ambulatory care on interpersonal aspects, such as responsiveness, communication, hospitality and being more client-orientated [2,46].

Our participants rated public hospital lower than birth centres and private hospitals in some essential facilities including clean environment, water and adequate rooms. Similar perceptions of lower quality about human and physical resources (bed, toilet, space) of public facilities were observed in India [3,47], and in antenatal care in Gambia [48]. In this study, the Nepalese women noticed the lack of equipment and competent health personnel in rural birth centres, in a similar fashion to Vietnamese women who responded negatively to equipment, resources and competency of health staff in commune health centres [33].

It is known that negative attitude of service providers can affect the use of professional midwifery care in Nepal [49]. Our findings on the low ratings of public hospital concerning interpersonal aspects support this notion. Experience of physical and verbal abuses, disrespectful treatment, and lack of attention were documented by women in India [3], Guatemala [50], Dominican Republic [51], and Cambodia [23]. On the other hand, that the perceived quality of the public hospital improved after delivery suggested maternity care was not as bad as expected. The reasons behind service provider attitudes are complex and multi-faceted in Nepal [35,49]. Maternity wards in public hospital are often overcrowded with high bed occupancy throughout the year [52]. The limited staff available have difficulty coping with the heavy workload while striving to assure the self-respect, dignity and professional care of the pregnant women, which may partly explain the poor interpersonal performance of public hospitals.

The private sector provides a substantial proportion of ambulatory care in developing countries [46]. However, for services such as immunisation, antenatal and delivery care, the role of private sector is more circumscribed [53]. In this study, despite the lower perceived quality on interpersonal aspects and essential facilities, the public hospital contributed the majority of delivery cases (76%), even though the private hospitals also took part in the government’s safer mother programme which provides free maternity services. Reasons underlying the high utilisation of the public hospital include its reputation and perceived higher technical quality than birth centres. Indeed, many rural women bypassed their nearest birth centre to deliver at the public hospital [44]. Birth centres should be supplied with competent midwives and more equipment, in order to convince women that they are capable of providing emergency obstetric care. They should be linked with the hospital by reliable transportation in case of an emergency. There is also an urgent need to improve essential facilities such as water supply, toilet and waiting room at the public hospital, as long as the provision of maternity services remains the responsibility of the government.

Several issues and limitations should be considered. There is a general tendency of courtesy bias from respondents in evaluating quality from the user’s perspective, especially in exit interviews [29]. Similarly, courtesy bias could not be ruled out in our household interviews. Although usage of a self-completed questionnaire might incur less courtesy bias, its administration would require an effective distribution system and educated respondents to avoid misinterpretation of the questions. In this study, comparison of perceived quality between the three types of facility was based on opinions from different groups of women, rather than ratings of facilities by the same individual. Nevertheless, these women were likely to be exposed to all types of facility, at least for different health seeking purposes or heard from others who have experienced such facilities.

Because our participants came from different ethnicities, the national Nepali language was used to validate the questionnaire and to conduct the interviews. Inclusion of women who delivered at home could have provided important clues on their non-utilisation of delivery services. However, they might be reluctant to give their opinions because of lack of experience using the maternity services. Finally, the quantitative nature of the survey methodology has potential shortcomings, because women may hold a complex set of experiences in labour and childbirth that could not be simply explained using a structured instrument alone. Some qualitative exploration might provide additional information to supplement the observed perceptions of quality.

## Conclusions

Overall, the perceptions of quality differed significantly by types of health facility used by women for delivery. They rated lowest the supplies and equipment in birth centres and the amenities and interpersonal aspects in the public hospital. Policy makers should consider providing more equipment and human resources to birth centres, training of public hospital staff on communications and interpersonal skills, and increasing the role of private hospitals in maternity service delivery.

## Competing interests

The authors declare that they have no competing interests.

## Authors’ contributions

RK conceived and managed the project and data collection, performed statistical analysis and drafted the manuscript. AHL contributed to the study design, data analysis and revision of the manuscript. PKP contributed to the project management and revision of the manuscript. All authors read and approved the final manuscript.

## Pre-publication history

The pre-publication history for this paper can be accessed here:

http://www.biomedcentral.com/1471-2393/14/45/prepub

## Supplementary Material

Additional file 1: Table S1 Questions on perceived quality of maternity services. Click here for file

Additional file 2: Table S2 Tukey’s post hoc comparison of perceived quality scale items between facility types. Click here for file

Additional file 3: Table S3 Multiple linear regression results for total perceived quality score and subscales. Click here for file
