# Supplementary material for: Women’s perception of quality of maternity services: a longitudinal survey in Nepal
Source: BMC Pregnancy Childbirth. 2014 Jan 24;14:45. doi: 10.1186/1471-2393-14-45 (PMC3902186; doi:10.1186/1471-2393-14-45)
Supplement: Additional file 1: Table S1 — Questions on perceived quality of maternity services. [file 1471-2393-14-45-S1.docx]

**Additional file: Table S1 – Questions on perceived quality of maternity services.**

| **Health Facility** | | | | |
| --- | --- | --- | --- | --- |
| In your opinion, the number of health staff in the health facility is **adequate.** | 1. Completely Disagree | 2. Disagree | 3. Agree | 4. Completely Agree |
| In your opinion, the health staffs in the health facility are **well suited** to treat women’s health problems. | 1. Completely Disagree | 2. Disagree | 3. Agree | 4. Completely Agree |
| In your opinion, the waiting rooms, examination rooms and other rooms of the health facility are **adequate** for women’s health problems. | 1. Completely Disagree | 2. Disagree | 3. Agree | 4. Completely Agree |
| In your opinion, the provision of clean drinking water, hand washing facilities, and toilets for women in the facility are **adequate.** | 1. Completely Disagree | 2. Disagree | 3. Agree | 4. Completely Agree |
| In your opinion, the overall environment of the health facility is **very clean**. | 1. Completely Disagree | 2. Disagree | 3. Agree | 4. Completely Agree |
| In your opinion, the equipment in the health facility is **well suited** for detecting women’s health problems. | 1. Completely Disagree | 2. Disagree | 3. Agree | 4. Completely Agree |
| The distance from your home to the health facility is **very far.** | 1. Completely Disagree | 2. Disagree | 3. Agree | 4. Completely Agree |
| **Health Care Delivery** | | | | |
| In your opinion, the health staff in the health facility examines pregnant and postpartum women **well**. | 1. Completely Disagree | 2. Disagree | 3. Agree | 4. Completely Agree |
| In your opinion, the health staffs in the health facility are **very capable** of finding out what is wrong with the patients. | 1. Completely Disagree | 2. Disagree | 3. Agree | 4. Completely Agree |
| In your opinion, the health staffs in the health facility prescribe the **drugs that are needed**. | 1. Completely Disagree | 2. Disagree | 3. Agree | 4. Completely Agree |
| In your opinion, the drugs supplied by this health facility are **good.** | 1. Completely Disagree | 2. Disagree | 3. Agree | 4. Completely Agree |
| In your opinion, patients can obtain drugs from this health facility **easily**. | 1. Completely Disagree | 2. Disagree | 3. Agree | 4. Completely Agree |
| The health facility provided **very much privacy** during vaginal examination and delivery. | 1. Completely Disagree | 2. Disagree | 3. Agree | 4. Completely Agree |
| You feel **very much** unnecessary and humiliating procedures during antenatal and delivery care. | 1. Completely Disagree | 2. Disagree | 3. Agree | 4. Completely Agree |
| In your opinion, the information of danger signs of delivery and postpartum provided by health staff is **adequate.** | 1. Completely Disagree | 2. Disagree | 3. Agree | 4. Completely Agree |
| **Interpersonal Aspects** | | | | |
| In your opinion, the health staffs in the health centre are **very open** with the patients. | 1. Completely Disagree | 2. Disagree | 3. Agree | 4. Completely Agree |
| In your opinion, the health staffs in the health centre are **very compassionate** towards the patients. | 1. Completely Disagree | 2. Disagree | 3. Agree | 4. Completely Agree |
| In your opinion, the health staffs are **respectful** towards the patients | 1. Completely Disagree | 2. Disagree | 3. Agree | 4. Completely Agree |
| In your opinion, the time that the health staffs devote to their patients is **adequate**. | 1. Completely Disagree | 2. Disagree | 3. Agree | 4. Completely Agree |
| In your opinion, the people in the health facility are **very honest.** | 1. Completely Disagree | 2. Disagree | 3. Agree | 4. Completely Agree |
| **Overall** | | | | |
| You were **completely satisfied** with the services provided to you | 1. Completely Disagree | 2. Disagree | 3. Agree | 4. Completely Agree |
| In the case of your **future delivery** or next baby, will you again use the health care facility? | yes | No | Undecided/do not know |  |
