# Supplementary material for: Women’s perception of quality of maternity services: a longitudinal survey in Nepal
Source: BMC Pregnancy Childbirth. 2014 Jan 24;14:45. doi: 10.1186/1471-2393-14-45 (PMC3902186; doi:10.1186/1471-2393-14-45)
Supplement: Additional file 2: Table S2 — Tukey’s post hoc comparison of perceived quality scale items between facility types. [file 1471-2393-14-45-S2.docx]

Additional file: Table S2 - Tukey’s post hoc comparison of perceived quality scale items between facility types.

| **Scale items** | **Type of facility** | **Type of facility** | **Mean difference** | **Standard error** | **P** |
| --- | --- | --- | --- | --- | --- |
| Adequacy of health staff | Public hospital | Private hospital | -0.23 | 0.09 | 0.033* |
|  |  | Birth centre | 0.39 | 0.07 | < 0.001* |
|  | Private hospital | Public hospital | 0.23 | 0.09 | 0.033* |
|  |  | Birth centre | 0.62 | 0.11 | < 0.001* |
|  | Birth centre | Public hospital | -0.39 | 0.07 | < 0.001* |
|  |  | Private hospital | -0.62 | 0.11 | < 0.001* |
| Health staff suited to women’s health | Public hospital | Private hospital | -0.29 | 0.09 | 0.003* |
|  |  | Birth centre | 0.41 | 0.07 | < 0.001* |
|  | Private hospital | Public hospital | 0.29 | 0.09 | 0.003* |
|  |  | Birth centre | 0.70 | 0.10 | < 0.001* |
|  | Birth centre | Public hospital | -0.41 | 0.07 | < 0.001* |
|  |  | Private hospital | -0.70 | 0.10 | < 0.001* |
| Adequacy of room | Public hospital | Private hospital | -0.77 | 0.09 | < 0.001* |
|  |  | Birth centre | -0.46 | 0.08 | < 0.001* |
|  | Private hospital | Public hospital | 0.77 | 0.09 | < 0.001* |
|  |  | Birth centre | 0.30 | 0.12 | 0.030* |
|  | Birth centre | Public hospital | 0.46 | 0.08 | < 0.001* |
|  |  | Private hospital | -0.30 | 0.12 | 0.030* |
| Adequacy of water | Public hospital | Private hospital | -0.85 | 0.10 | < 0.001* |
|  |  | Birth centre | -0.29 | 0.08 | 0.002* |
|  | Private hospital | Public hospital | 0.85 | 0.10 | < 0.001* |
|  |  | Birth centre | 0.55 | 0.12 | < 0.001* |
|  | Birth centre | Public hospital | 0.29 | 0.08 | 0.002* |
|  |  | Private hospital | -0.55 | 0.12 | < 0.001* |
| Environment clean | Public hospital | Private hospital | -0.94 | 0.10 | < 0.001* |
|  |  | Birth centre | -0.43 | 0.08 | < 0.001* |
|  | Private hospital | Public hospital | 0.94 | 0.10 | < 0.001* |
|  |  | Birth centre | 0.51 | 0.12 | < 0.001* |
|  | Birth centre | Public hospital | 0.43 | 0.08 | < 0.001* |
|  |  | Private hospital | -0.51 | 0.12 | < 0.001* |
| Adequacy of medical equipment | Public hospital | Private hospital | -0.42 | 0.08 | < 0.001* |
|  |  | Birth centre | 0.41 | 0.07 | < 0.001* |
|  | Private hospital | Public hospital | 0.42 | 0.08 | < 0.001* |
|  |  | Birth centre | 0.84 | 0.10 | < 0.001* |
|  | Birth centre | Public hospital | -0.41 | 0.07 | < 0.001* |
|  |  | Private hospital | -0.84 | 0.10 | < 0.001* |
| Distance | Public hospital | Private hospital | -0.24 | 0.09 | 0.019* |
|  |  | Birth centre | -0.02 | 0.07 | 0.932 |
|  | Private hospital | Public hospital | 0.24 | 0.09 | 0.019* |
|  |  | Birth centre | 0.21 | 0.10 | 0.116 |
|  | Birth centre | Public hospital | 0.02 | 0.07 | 0.932 |
|  |  | Private hospital | -0.21 | 0.10 | 0.116 |
| Examine well | Public hospital | Private hospital | -0.31 | 0.08 | 0.001* |
|  |  | Birth centre | 0.14 | 0.07 | 0.118 |
|  | Private hospital | Public hospital | 0.31 | 0.08 | 0.001* |
|  |  | Birth centre | 0.45 | 0.10 | < 0.001* |
|  | Birth centre | Public hospital | -0.14 | 0.07 | 0.118 |
|  |  | Private hospital | -0.45 | 0.10 | < 0.001* |
| Staff capable | Public hospital | Private hospital | -0.35 | 0.08 | < 0.001* |
|  |  | Birth centre | 0.17 | 0.06 | 0.025* |
|  | Private hospital | Public hospital | 0.35 | 0.08 | < 0.001* |
|  |  | Birth centre | 0.52 | 0.09 | < 0.001* |
|  | Birth centre | Public hospital | -0.17 | 0.06 | 0.025* |
|  |  | Private hospital | -0.52 | 0.09 | < 0.001* |
| Prescription of drugs | Public hospital | Private hospital | -0.32 | 0.07 | < 0.001* |
|  |  | Birth centre | 0.01 | 0.06 | 0.962 |
|  | Private hospital | Public hospital | 0.32 | 0.07 | < 0.001* |
|  |  | Birth centre | 0.33 | 0.09 | 0.001* |
|  | Birth centre | Public hospital | -0.01 | 0.06 | 0.962 |
|  |  | Private hospital | -0.33 | 0.09 | 0.001* |
| Quality of drugs | Public hospital | Private hospital | -0.25 | 0.07 | 0.003* |
|  |  | Birth centre | 0.15 | 0.06 | 0.039* |
|  | Private hospital | Public hospital | 0.25 | 0.07 | 0.003* |
|  |  | Birth centre | 0.41 | 0.09 | < 0.001* |
|  | Birth centre | Public hospital | -0.15 | 0.06 | 0.039* |
|  |  | Private hospital | -0.41 | 0.09 | < 0.001* |
| Availability of drugs | Public hospital | Private hospital | -0.24 | 0.08 | 0.013* |
|  |  | Birth centre | 0.09 | 0.07 | 0.399 |
|  | Private hospital | Public hospital | 0.24 | 0.08 | 0.013* |
|  |  | Birth centre | 0.34 | 0.10 | 0.004* |
|  | Birth centre | Public hospital | -0.09 | 0.07 | 0.399 |
|  |  | Private hospital | -0.34 | 0.10 | 0.004* |
| Privacy | Public hospital | Private hospital | -0.47 | 0.09 | < 0.001* |
|  |  | Birth centre | -0.08 | 0.07 | 0.509 |
|  | Private hospital | Public hospital | 0.47 | 0.09 | < 0.001* |
|  |  | Birth centre | 0.39 | 0.11 | 0.003* |
|  | Birth centre | Public hospital | 0.08 | 0.07 | 0.509 |
|  |  | Private hospital | -0.39 | 0.11 | 0.003 |
| Unnecessary act | Public hospital | Private hospital | -0.03 | 0.04 | 0.708 |
|  |  | Birth centre | -0.05 | 0.03 | 0.336 |
|  | Private hospital | Public hospital | 0.03 | 0.04 | 0.708 |
|  |  | Birth centre | -0.01 | 0.05 | 0.949 |
|  | Birth centre | Public hospital | 0.05 | 0.03 | 0.336 |
|  |  | Private hospital | 0.01 | 0.05 | 0.949 |
| Adequacy of information | Public hospital | Private hospital | -0.45 | 0.08 | < 0.001* |
|  |  | Birth centre | -0.03 | 0.07 | 0.904 |
|  | Private hospital | Public hospital | 0.45 | 0.08 | < 0.001* |
|  |  | Birth centre | 0.42 | 0.10 | < 0.001* |
|  | Birth centre | Public hospital | 0.03 | 0.07 | 0.904 |
|  |  | Private hospital | -0.42 | 0.10 | < 0.001* |
| Openness to patients | Public hospital | Private hospital | -0.43 | 0.09 | < 0.001* |
|  |  | Birth centre | -0.23 | 0.07 | 0.008* |
|  | Private hospital | Public hospital | 0.43 | 0.09 | < 0.001* |
|  |  | Birth centre | 0.20 | 0.11 | 0.159 |
|  | Birth centre | Public hospital | 0.23 | 0.07 | 0.008* |
|  |  | Private hospital | -0.20 | 0.11 | 0.159 |
| Compassion for patients | Public hospital | Private hospital | -0.39 | 0.09 | < 0.001* |
|  |  | Birth centre | -0.30 | 0.07 | < 0.001* |
|  | Private hospital | Public hospital | 0.39 | 0.09 | < 0.001* |
|  |  | Birth centre | 0.08 | 0.11 | 0.698 |
|  | Birth centre | Public hospital | 0.30 | 0.07 | < 0.001* |
|  |  | Private hospital | -0.08 | 0.11 | 0.698 |
| Respect for patients | Public hospital | Private hospital | -0.41 | 0.08 | < 0.001* |
|  |  | Birth centre | -0.39 | 0.07 | < 0.001* |
|  | Private hospital | Public hospital | 0.41 | 0.08 | < 0.001* |
|  |  | Birth centre | 0.01 | 0.10 | 0.983 |
|  | Birth centre | Public hospital | 0.39 | 0.07 | < 0.001* |
|  |  | Private hospital | -0.01 | 0.10 | 0.983 |
| Time devoted to patients | Public hospital | Private hospital | -0.52 | 0.08 | < 0.001* |
|  |  | Birth centre | -0.25 | 0.06 | < 0.001* |
|  | Private hospital | Public hospital | 0.52 | 0.08 | < 0.001* |
|  |  | Birth centre | 0.27 | 0.09 | 0.015* |
|  | Birth centre | Public hospital | 0.25 | 0.06 | < 0.001* |
|  |  | Private hospital | -0.27 | 0.09 | 0.015* |
| Honesty | Public hospital | Private hospital | -0.30 | 0.07 | < 0.001* |
|  |  | Birth centre | -0.08 | 0.06 | 0.376 |
|  | Private hospital | Public hospital | 0.30 | 0.07 | < 0.001* |
|  |  | Birth centre | 0.22 | 0.09 | 0.055 |
|  | Birth centre | Public hospital | 0.08 | 0.06 | 0.376* |
|  |  | Private hospital | -0.22 | 0.09 | 0.055 |
| **Health facility** | Public hospital | Private hospital | -3.78 | 0.48 | < 0.001* |
|  |  | Birth centre | -0.01 | 0.40 | < 0.001 |
|  | Private hospital | Public hospital | 3.78 | 0.48 | < 0.001* |
|  |  | Birth centre | 3.76 | 0.59 | < 0.001* |
|  | Birth centre | Public hospital | 0.01 | 0.40 | < 0.001 |
|  |  | Private hospital | -3.76 | 0.59 | < 0.001* |
| **Health care delivery** | Public hospital | Private hospital | -2.45 | 0.46 | < 0.001* |
|  |  | Birth centre | 0.40 | 0.38 | 0.544 |
|  | Private hospital | Public hospital | 2.45 | 0.46 | < 0.001* |
|  |  | Birth centre | 2.86 | 0.56 | < 0.001* |
|  | Birth centre | Public hospital | -0.40 | 0.38 | 0.544 |
|  |  | Private hospital | -2.86 | 0.56 | < 0.001* |
| **Interpersonal aspects** | Public hospital | Private hospital | -2.08 | 0.37 | < 0.001* |
|  |  | Birth centre | -1.27 | 0.30 | < 0.001* |
|  | Private hospital | Public hospital | 2.08 | 0.37 | < 0.001* |
|  |  | Birth centre | 0.80 | 0.45 | 0.172 |
|  | Birth centre | Public hospital | 1.27 | 0.30 | < 0.001* |
|  |  | Private hospital | -0.80 | 0.45 | 0.172 |
| **Total score** | Public hospital | Private hospital | -8.33 | 1.16 | < 0.001* |
|  |  | Birth centre | -0.88 | 0.96 | 0.629 |
|  | Private hospital | Public hospital | 8.33 | 1.16 | < 0.001* |
|  |  | Birth centre | 7.44 | 1.41 | < 0.001* |
|  | Birth centre | Public hospital | 0.88 | 0.96 | 0.629 |
|  |  | Private hospital | -7.44 | 1.41 | < 0.001* |

*P < 0.05
