# Supplementary material for: Women’s perception of quality of maternity services: a longitudinal survey in Nepal
Source: BMC Pregnancy Childbirth. 2014 Jan 24;14:45. doi: 10.1186/1471-2393-14-45 (PMC3902186; doi:10.1186/1471-2393-14-45)
Supplement: Additional file 3: Table S3 — Multiple linear regression results for total perceived quality score and subscales. [file 1471-2393-14-45-S3.docx]

**Additional file: Table S3 - Multiple linear regression results for total perceived quality score and subscales.**

| **Type of health facility** | **Health facility** | **Health care delivery** | **Interpersonal aspects** | **Total score** |
| --- | --- | --- | --- | --- |
|  | Coefficient ^#^ (standard error) | Coefficient ^#^ (standard error) | Coefficient ^#^ (standard error) | Coefficient ^#^ (standard error) |
| Birth centre | Reference | Reference | Reference | Reference |
| Public hospital | -0.56 (0.45) | 0.02 (0.44) | -1.39* (0.35) | -1.93 (1.10) |
| Private hospital | 3.08* (0.62) | 2.46* (0.61) | 0.74 (0.48) | 6.30* (1.50) |

* P < 0.001

^#^ Adjusted for age, parity, wealth, caste, education level and distance to reach facility
